# Independent role of two novel abdominal fat indicators in chronic diarrhoea

**DOI:** 10.3389/fmed.2025.1593571

**Published:** 2025-07-29

**Authors:** Shiming Chen, Jingxia Zhang, Yingxue Niu, Yifang Li

**Affiliations:** ^1^College of Nursing, Anhui University of Chinese Medicine, Hefei, China; ^2^Laboratory of Geriatric Nursing and Health, Anhui University of Chinese Medicine, Hefei, China

**Keywords:** body shape index, body roundness index, chronic diarrhoea, NHANES, cross-sectionaI study

## Abstract

**Background:**

Studies have confirmed that obesity is an antecedent of chronic diarrhoea, and new evidence suggests that visceral fat accumulation may play a more critical role than total body fat in intestinal dysfunction and the development of chronic diarrhoea. Traditional body mass index (BMI) does not accurately reflect fat distribution, limiting the depth of relevant research. Body shape index (ABSI) and body rounding index (BRI), as emerging measurements that more accurately assess abdominal and visceral adiposity, have shown superior predictive value to BMI in cardiovascular and metabolic diseases. However, the use of these metrics in the prediction of chronic diarrhoea has not been explored. This study is the first to investigate the relationship between ABSI and BRI and chronic diarrhoea, aiming to provide a new clinical tool for risk assessment of obesity-related diarrhoea.

**Methods:**

This study used data from the Bowel Health Questionnaire (BHQ) of the U.S. National Health Examination Survey (NHANES) database (2005–2010), and chronic diarrhoea was defined as “chronic diarrhoea” by the Bristol Stool Scale (BSFS) types 6 andI7(4). Weighted logistic regression and trend analyses were performed to examine the association between ABSI/BRI and chronic diarrhoea. Flexible restricted cubic spline (RCS) models showed dynamic associations. Stratified analyses examined associations between age, gender, race, and clinical characteristics (e.g., cardiovascular disease, diabetes, hypertension). Receiver operating characteristic (ROC) curves assessed the predictive performance of ABSI/BRI for risk of chronic diarrhoea.

**Results:**

Multivariate regression models with time-trend analyses indicated a dose–response relationship between higher BRI percentiles and the incidence of chronic diarrhoea. 13% per unit increase in BRI (OR = 1.13, 95% CI = 1.08–1.19, *p* < 0.001). Similarly, there was a 35% increase in risk for each 0.01 unit increase in ABSI (OR = 1.35, 95% CI = 1.01–1.80, *p* = 0.045), suggesting that those with higher ABSI were at higher risk. Subgroup analyses showed no significant interaction effect between BRI and chronic diarrhoea across age, sex, race, cardiovascular disease, diabetes, and hypertension (*p* > 0.050). The ROC confirmed the nonlinear association between ABSI/BRI and chronic diarrhoea.

**Conclusion:**

The objective of this study was to investigate the association between two novel abdominal fat indicators (ABSI and BRI) and chronic diarrhoea using nationally representative NHANES data (2005–2010). For the first time, we have identified ABSI and BRI as potentially useful clinical predictors of chronic diarrhoea.

## 1 Introduction

Chronic diarrhoea was defined as predominantly loose stools lasting more than 4 weeks (1). Chronic diarrhoea affects 17–30% of the global population (2), with significantly higher prevalence (21–30%) observed in obese individuals (3). Christopher Ma et al. found that chronic diarrhoea results in approximately 700,000 U.S. outpatient visits per year, costing $690 million annually (4). Chronic diarrhoea therefore not only poses a threat to patients’ health, but also creates a huge financial burden and reduces their quality of life. Obesity is a global public health problem, and regarding the causal relationship between obesity and chronic diarrhoea, current research suggests that obesity may be an antecedent to chronic diarrhoea (2, 5–7). It has been shown that bile acid malabsorption diarrhoea and secretory diarrhoea predominate in obese patients (6, 8). Firstly, obesity alters the composition of the intestinal bacterial flora and increases intestinal permeability, allowing bacterial endotoxins (e.g., lipopolysaccharides, LPS) to enter the bloodstream, which promotes intestinal permeability specialists to trigger diarrhoea (8). Secondly, obesity may accelerate the transit time in the large intestine, which leads to a shorter retention time of food and waste in the intestines, which can trigger diarrhoea (5, 8–11).

Research suggests that visceral fat accumulation may play a more critical role than total body fat in the development of intestinal dysfunction and chronic diarrhoea (12), and that the inability of body mass index (BMI) to accurately reflect fat distribution limits the depth of relevant studies (13–16). The ABSI is a new anthropometric tool that combines waist circumference, weight and height (17). It can provide an improved ability to quantify visceral and abdominal fat, which are key contributors to metabolic risk. The index was originally developed by Krakauer et al. in 2012 based on the National Health and Nutrition Examination Survey (NHANES) database (18, 19), correlation with visceral adiposity and metabolic risk has been validated in multiple population-based cohorts in the United States, Europe, and Asia (18–20). Developed by Thomas and colleagues in 2013, the BRI is a geometric metric based on the human ellipse approximation (21), the BRI recognizes individuals with the same BMI but different body fat distributions, and its validity has been verified in a database and imaging MRI technique at the University of Kiel, Germany, and in large cohort studies in several countries (22–25). Although not yet routinely used in clinical practice, these two indices have significant practical advantages: they can be quickly calculated from standardized formulas using only three parameters, namely waist circumference, height and weight, obtained during a routine physical examination, without the need for additional equipment or complex tests. This non-invasive feature makes it particularly suitable for large-scale epidemiological studies and risk screening in primary care settings, compared to fat quantification methods that require expensive imaging.

## 2 Materials and methods

### 2.1 Study design and participant selection from NHANES 2005–2010

**Figure 1:**
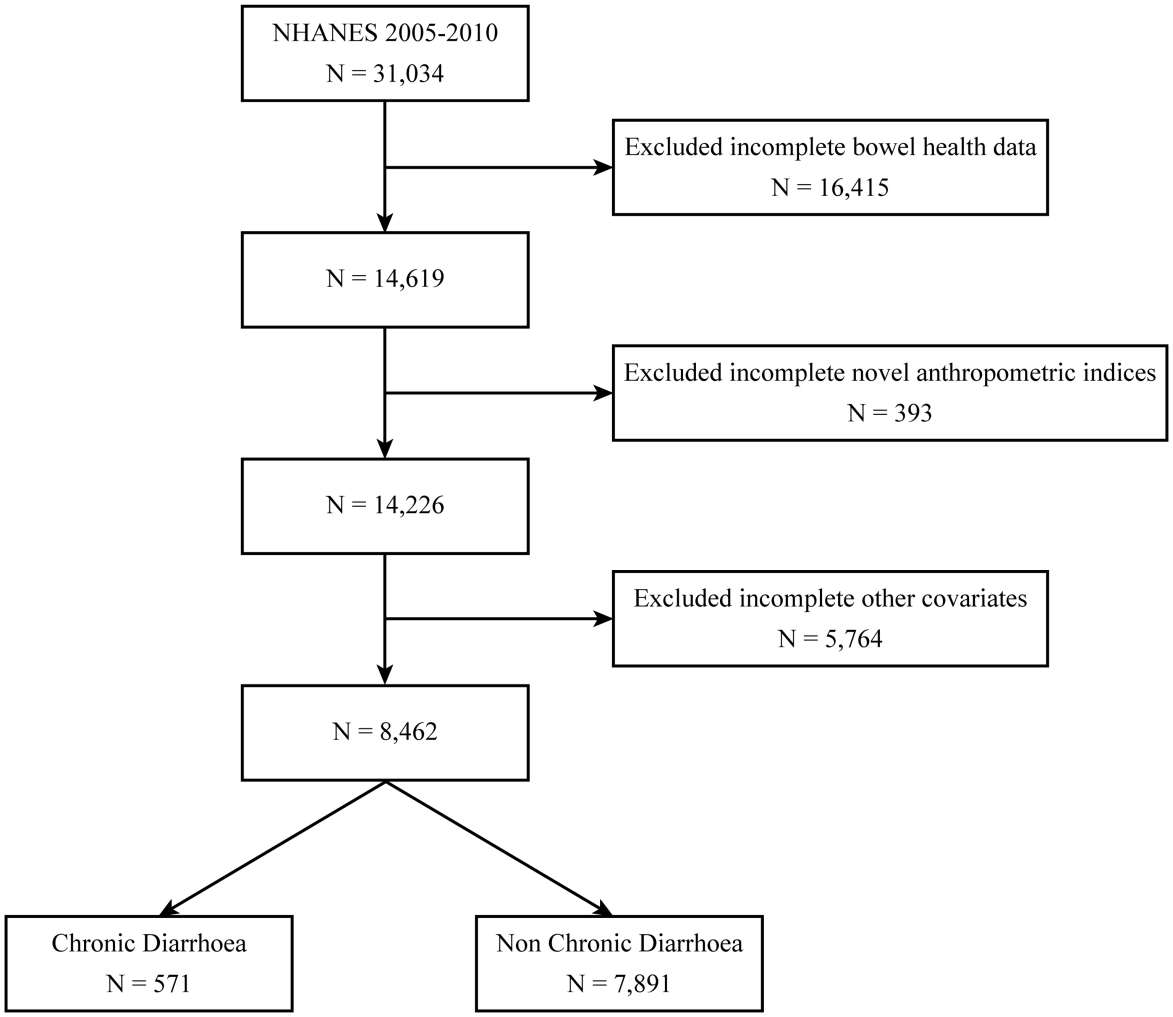
Flow chart of the screening of the NHANES 2005–2010 participants.

NHANES is a cross-sectional study that provides a nationally representative snapshot of the health status of the non-institutionalized population in the United States. From 2005 through 2010, the nationally representative NHANES dataset served as the epidemiological foundation for this cross-sectional investigation, initially comprising 31,034 participants. After excluding 16,415 individuals with incomplete bowel health questionnaire responses, 14,619 participants remained. Subsequent removal of 393 cases lacking novel anthropometric indices (ABSI/BRI) yielded 14,226 eligible subjects. After excluding 5,764 participants with missing covariate data, the final cohort consisted of 8,462 individuals, stratified into two groups: chronic diarrhoea (*n* = 571) and non-chronic diarrhoea (*n* = 7,891) (Figure 1). Ethical clearance for the research protocol was formally granted through the institutional review mechanisms of the National Center for Health Statistics Ethics Review Board (13). The NHANES website^1^ (23) offers comprehensive details on survey design, methodologies, population demographics, and dataset access. RetryClaude does not have internet access. Links provided may not be accurate or up to date. Claude can make mistakes. Please double-check responses.

### 2.2 Bowel health questionnaire

The bowel health questionnaire captured self-reported stool characteristics based on standardized classifications. Stool consistency was categorized into Types 1–7 following the Bristol Stool Form Scale (BSFS), a clinically validated diagnostic tool for fecal consistency assessment and morphological characterization. This scale operationally defines stool characteristics along a continuum: Type 1 (separate hard lumps, nut-like) to Type 7 (watery stools without solid fragments). Chronic diarrhoea was defined as persistent passage of Types 6 or 7 stools, while Types 1–2 indicated chronic constipation. Types 3–5 represented normal bowel patterns (26).

### 2.3 Covariate definitions and measurement protocols

Analyses adjusted for: age; sex (male ref); race/ethnicity (Mexican American, non-Hispanic Black, non-Hispanic White, other); education (<high school, high school, ≥college); marital status (married/cohabiting, single); alcohol use (heavy, low/moderate, non-drinker); smoking (current, former, never); PIR (<1.30, 1.30–3.49, ≥3.50); diabetes (defined by ≥1 of: FPG ≥ 7.0 mmol/L, OGTT>11.1 mmol/L, random glucose>11.1 mmol/L, HbA1c > 6.5%, diabetes medication use, or self-reported diagnosis); hypertension; CVD: Diagnosis ofCVD was determined by self-reported physician diagnoses obtained during individual interviews using a standardized medical condition questionnaire. Participants were asked: “Has a doctor or other health professional ever told you that you have congestive heart failure/coronary heart disease/angina/myocardial infarction/stroke?” A person was considered to have CVD if they answered “yes” to any of the above questions. Congestive heart failure, myocardial infarction, angina pectoris, and coronary artery disease were also defined according to the questions for the respective diseases listed above (27). Hypertension: The blood pressure measurement protocol follows the procedures established by the American Heart Association. After 3 measurements of blood pressure at rest, the average of systolic blood pressure (SBP) and diastolic blood pressure (DBP) was calculated. The 2017 American Heart Association/American College of Cardiology (AHA/ACC) guidelines recommend that individuals with an SBP ≥ 130 mmHg and/or a DBP ≥ 80 mmHg should be defined as hypertensive. In addition, participants who answered “yes” to the question “Have ou ever been told you have hypertension?” were categorized as hypertensive (28).

### 2.4 Calculation of body shape index (ABSI) and body roundness index (BRI)

Standardized anthropometric protocols were implemented to obtain iliac crest-level waist circumference measurements, along with standing height and body weight recordings. The following formulas were used to calculate BRI and ABSI:

BRI = 364.2-365.5 × √ 1-[(WC (cm)/2π)/(0.5 ×height (cm))]^2^ (21)

ABSI = WC (cm)/(BMI (kg/m^2^)^2/3^ × height (cm)^1/2^ (19)

### 2.5 Statistical analysis

Several statistical approaches were applied to examine the relationships between ABSI/BRI and chronic diarrhoea. For descriptive statistics, Continuous variables following a normal distribution were expressed as mean ± standard deviation, whereas categorical variables were reported as percentages. Differences between groups were evaluated using independent tests for continuous variables and chi-square tests for categorical variables. Appropriate sampling weights were applied to ensure national representativenes. ABSI and BRI were categorized into quartiles to better characterize population subgroups. We conducted weighted logistic regression analyses to evaluate ABSI/BRI-diarrhoea associations across progressive covariate adjustment levels: Model 1 (unadjusted), Model 2 (partially adjusted with demographics: age, sex, race, poverty-income ratio, and marital status), and Model 3 (fully adjusted for age, sex, race, poverty-income ratio, marital status, education, smoking, alcohol consumption, hypertension, diabetes, cardiovascular disease, total cholesterol, triglycerides, hemoglobin, and HbA1c) (29). Restricted cubic spline (RCS) curves with four knots were used to capture potential nonlinear relationships between anthropometric indices and chronic diarrhoea. Additionally, we performed stratified analyses by age, sex, race, cardiovascular disease, diabetes, and hypertension to assess effect heterogeneity across different subgroups. Multiplicative interaction terms were included in regression models to determine statistical significance (*p* < 0.05) for differences between subgroups (30). R4.3.1^2^ was used to perform all data analyses. A two-sided *p* < 0.05 was considered statistically significant.

The ROC curves were generated to evaluate the predictive performance of BRI, ABSI and traditional indices (body mass index, waist circumference, visceral adiposity index). Area under the curve (AUC) was used to quantify overall accuracy. Sensitivity and specificity were derived from the ROC curves at the optimal cutoff point determined by the Youden index (J = sensitivity + specificity − 1) (31).

## 3 Result

### 3.1 Demographic and clinical characteristics of study participants

A total of 8,462 participants were recruited for this study, of which 6.7% (571) were categorized in the chronic diarrhoea group and the remaining 93.3% (7891) in the non-chronic diarrhoea group. The chronic diarrhoea group was older (49.74 years vs. 46.11 years). The proportion of females was higher in the chronic diarrhoea group (51.3% versus 45.8%), *p* = 0.029. When examining BRI quartiles, a higher proportion of chronic diarrhoea patients were found in Q4 (36. 1%) compared to Q1 (17.3%), <0.05. Similarly, for ABSI quartiles, a higher proportion of chronic diarrhoea patients were observed in Q4 (33.8%) compared to Q (25.6%), *p* = 0.002. In terms of marital status, high blood pressure, cardiovascular disease (CVD), total cholesterol (TC), triglycerides (TG), and PIR (poverty-to-income ratio), *p* ≥ 0.050 (Table 1).

**Table 1:** Baseline characteristics of the study population analyzed based on chronic diarrhoea status.

Characteristics	No Chronic diarrhoea (*N* = 7,891)	Chronic diarrhoea (*N* = 571)	*p*-value
Age (years)	46.11 ± 17.11	49.74 ± 15.81	<0.001
Female	3,614 (45.8)	293 (51.3)	0.029
Race, *n* (%)			0.021
Mexican American	1,331 (16.9)	134 (23.5)	
Non-Hispanic Black	1,368 (17.3)	104 (18.2)	
Non-Hispanic White	4,284 (54.3)	263 (46. 1)	
Others	908 (11.5)	70 (12.3)	
Education level, *n* (%)			<0.001
<High school	1703 (21.6)	205 (35.9)	
High school	1832 (23.2)	122 (21.4)	
> High school	4,356 (55.2)	244 (42.7)	
Marital status, *n* (%)			0.920
Married/Cohabiting	4,950(62.7)	353 (61.8)	
Single^a^	2,941 (37.3)	218 (38.2)	
Drinking alcohol, *n* (%)			0.009
Heavy drinker	1,082 (13.7)	107 (18.7)	
Low to moderate drinker	5,995 (76.0)	399 (69.9)	
Non-drinker	814 (10.3)	65 (11.4)	
Smoke, *n* (%)			0.016
Current	2,008 (25.4)	177 (31.0)	
Former	2,073 (26.3)	150 (26.3)	
Never	3,810 (48.3)	244 (42.7)	
PIR, *n* (%)			0.183
<1.3	1,955 (24.8)	182 (31.9)	
≥3.5	3,040 (38.5)	181 (31.7)	
1.3 to<3.5	2,896 (36.7)	208 (36.4)	
Diabetes mellitus, *n* (%)	951 (12. 1)	93 (16.3)	0.042
Hypertension, *n* (%)	3,753 (47.6)	303 (53. 1)	0.641
CVD, *n* (%)	635 (8.0)	53 (9.3)	0.332
TC (mg/dl)	197.59 ± 41.62	200.54 ± 41.59	0.112
TG (mg/dl)	155.16 ± 131.44	162.18 ± 112.75	0.495
Hb (g/dl)	14.41 ± 1.49	14.17 ± 1.57	0.008
HbA1c (%)	5.56 ± 0.90	5.67 ± 1.02	0.030
BRI, *n* (%)			<0.001
Q1	2,017 (25.6)	99 (17.3)	
Q2	2,007 (25.4)	108 (18.9)	
Q3	1,957 (24.8)	158 (27.7)	
Q4	1,910 (24.2)	206 (36. 1)	
ABSI, *n* (%)			0.002
Q1	2,017 (25.6)	99 (17.3)	
Q2	1,982 (25. 1)	133 (23.3)	
Q3	1,969 (25.0)	146 (25.6)	
Q4	1,923 (24.4)	193 (33.8)	

Mean ±standard deviation for continuous variables, the P-value was calculated by the weighted linear regression model; *n* (%) for categorical variables, the *p*-value was calculated by the weighted chi-square test. Single^a^, Widowed/Divorced/Separated/Never married. PIR, poverty income ratio; CVD, cardiovascular disease; TC, total cholesterol; TG, triglycerides; Hb, hemoglobin; HbA1c, hemoglobin A1c; BRI, body roundness index; ABSI, a body shape index; Q1, first quartile; Q2, second quartile; Q3, third quartile; Q4, fourth quartile.

### 3.2 BRI and ABSI and the risk of chronic diarrhoea

According to Table 2, Higher BRI and ABSI were significantly linked to an increased risk of chronic diarrhoea. In the unadjusted model (Model 1), each 1-unit increase in BRI corresponded to a 12% higher risk of chronic diarrhoea (OR = 1.12, 95% CI: 1.08–1.17, *p* < 0.001). This association persisted in the fully adjusted model (Model 3), with a 13% risk increase per BRI unit (OR = 1.13, 95% CI 1.08–1.19, *p* < 0.001). Quartile analysis further revealed that participants in the highest BRI quartile (Q4) had 1.91-fold greater odds of chronic diarrhoea compared to the lowest quartile (Q1) (OR = 1.91, 95% CI: 1.29–2.83, *p* = 0.002). Similarly, every 0.01-unit increment in ABSI demonstrated a statistically significant correlation with increased risk of chronic diarrhoea. In the unadjusted model (Model 1), ABSI demonstrated a strong positive association (OR = 1.53, 95% CI: 1.19–1.95, *p* = 0.001). This association remained significant after full covariate adjustment in Model 3 (OR = 1.35, 95% CI: 1.01–1.80, *p* = 0.045). Participants in the highest ABSI quartile (Q4) exhibited a 1.67-fold increased risk compared to the lowest quartile (Q1) (OR = 1.67, 95% CI: 1.12–2.48, *p* = 0.014). These results collectively indicate that both BRI and ABSI are independently associated with chronic diarrhoea risk.

**Table 2:** Body measures (BRI and ABSI) and chronic diarrhoea.

Variable	Model 1^a^ OR (95% CI) *p*-value	Model 2^b^ OR (95% CI) *p*-value	Model 3^c^ OR (95% CI) *p*-value
BRI per 1 unit increase	1.12 (1.08–1.17) < 0.001	1.10 (1.06–1.14) < 0.001	1.13 (1.08–1.19) < 0.001
Quartile 1	1 [Reference]	1 [Reference]	1 [Reference]
Quartile 2	1.02 (0.73–1.42) 0.923	0.95 (0.68–1.34) 0.778	0.99 (0.69–1.42) 0.964
Quartile 3	1.35 (0.95–1.92) 0.090	1.20 (0.84–1.71) 0.296	1.31 (0.90–1.91) 0.145
Quartile 4	1.93 (1.42–2.62) < 0.001	1.65 (1.20–2.27) 0.003	1.91 (1.29–2.83) 0.002
*p* for trend	1.26 (1.15–1.39) < 0.001	1.20 (1.08–1.33) < 0.001	1.26 (1.11–1.42) < 0.001
ABSI per 0.1 unit increase	1.53 (1.19–1.95) 0.001	1.41 (1.05–1.89) 0.024	1.35 (1.01–1.80) 0.045
Quartile 1	1 [Reference]	1 [Reference]	1 [Reference]
Quartile 2	1.29 (0.88–1.88) 0.185	1.28 (0.87–1.90) 0.203	1.29 (0.86–1.92) 0.202
Quartile 3	1.36 (1.00–1.86) 0.052	1.31 (0.95–1.83) 0.100	1.29 (0.92–1.80) 0.133
Quartile 4	1.88 (1.33–2.66) < 0.001	1.74 (1.17–2.58) 0.008	1.67 (1.12–2.48) 0.014
*p* for trend	1.22 (1.10–1.35) < 0.001	1.18 (1.05–1.33) 0.008	1.16 (1.03–1.31) 0.015

^ᵃ^Model 1: No adjustment for covariates. ^ᵇ^Model 2: Adjusted for age, sex, race, PIR, marital status, education level and PIR. ^ᶜ^Model 3: Adjusted for smoking, alcohol use, hypertension, CVD, DM, TC, TG, Hb and HbA1c based on Model 2. BRI, Body Roundness Index; ABSI, The Body Shape Index.

**Figure 2:**
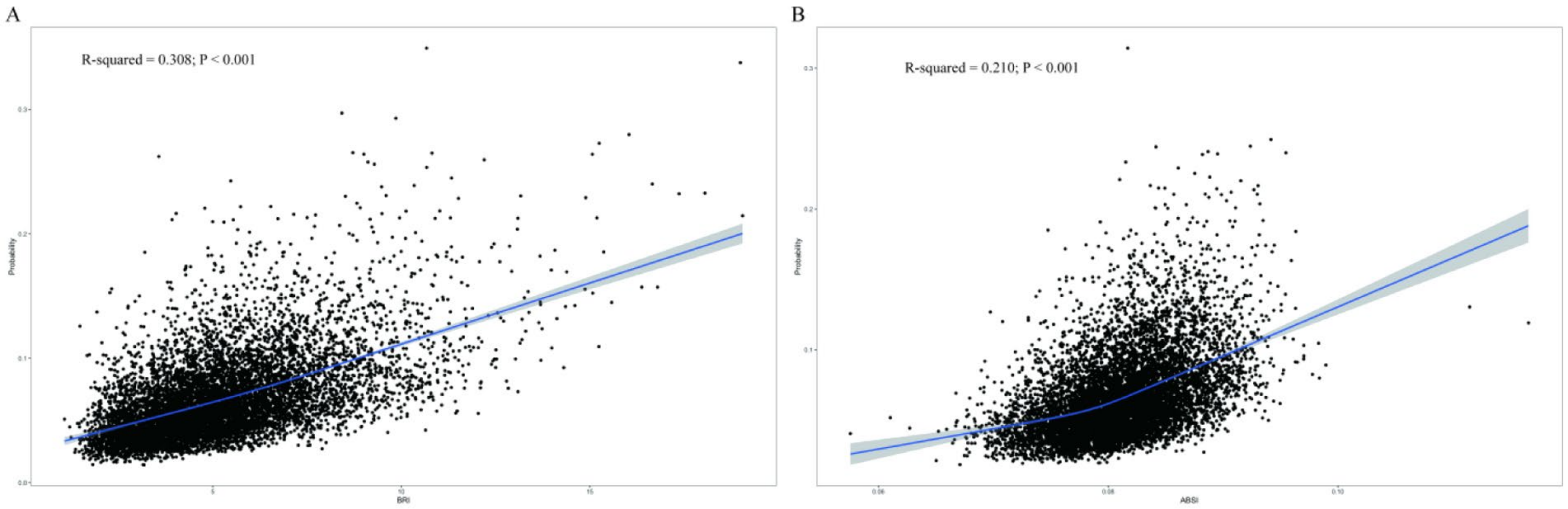
Restricted cubic spline curves for the relationship between the new anthropometric index and chronic diarrhoea. **(A)** BRI and **(B)** ABSI.

In the analysis of BRI (Figure 2A), the R-squared value of 0.308 (*p* < 0.001) indicated a significant relationship between BRI and the risk of chronic diarrhoea, with risk escalating progressively as BRI increased. For ABSI (Figure 2B), the R-squared value of 0.210 (*p* < 0.001) suggested a modest yet significant positive correlation with chronic diarrhoea. Although ABSI exhibited weaker predictive capacity compared to BRI, it still demonstrated clinical utility by identifying high-risk individuals, particularly males (OR = 2.76, 95% CI: 1.70–4.47), and showed consistent associations across different patient subgroups, making it a valuable complementary tool for risk assessment in clinical practice. Thus, BRI demonstrated superior predictive utility and explanatory power for chronic diarrhoea risk, whereas ABSI, despite its lower correlation, retained diagnostic relevance in risk stratification.

### 3.3 Subgroup analysis between BRI and ABSI and chronic diarrhoea

Analyses across subgroups of age, sex, race, cardiovascular disease, diabetes, and hypertension showed no statistically significant interaction effects (all *p* for interaction>0.05), demonstrating homogeneity in the BRI-chronic diarrhoea association across these stratified subgroups (Table 3). Stratified analyses showed significant ABSI-chronic diarrhoea associations in males (OR = 2.76, 95% CI:1.70–4.47, *p* < 0.001), indicating aged<60 years (OR = 1.50, 95% CI:1.04–2.15, *p* = 0.030), non-CVD (OR = 1.35, 95% CI:1.01–1.79, *p* = 0.041), and the hypertensive groups (OR = 1.65, 95% CI:1.10–2.46, *p* = 0.017), with a significant sex interaction (*p*-interaction = 0.009). The ABSI-diarrhoea link demonstrated stability across demographics, modified only by sex (Table 4).

**Table 3:** Subgroup analysis of the association between BRI and chronic diarrhoea.

Subgroups	Adjusted OR (95%CI)	*p*-value	*p* for interaction
Age (year)			0.251
<60 years	1.13 (1.07–1. 19)	<0.001	
≥60 years	1.14 (1.03–1. 27)	0.012	
Sex			0.737
Male	1. 15 (1.06–1.25)	0.002	
Female	1.11 (1.04–1. 19)	0.003	
Race			0.702
Mexican	1. 17 (1.05–1.31)	0.008	
American			
Non-Hispanic White	1.13 (1.05–1.22)	0.002	
Non-Hispanic Black	1. 15 (1.04–1.26)	0.010	
Others	1. 14 (0.99–1.32)	0.060	
CVD			0.096
Yes	1.00 (0.87–1. 14)	0.966	
No	1. 14 (1.09–1.20)	<0.001	
Diabetes			0.414
Yes	1.11 (0.99–1.23)	0.067	
No	1. 14 (1.08–1.21)	<0.001	
Hypertension			0.931
Yes	1. 14 (1.07–1.21)	<0.001	
No	1.13 (1.01–1.26)	0.029	

ABSI, a body shape index; OR, odds ratio; CI, confidence interval; CVD, cardiovascular disease.

**Table 4:** Analyses examining subgroups assessed the association between ABSI and chronic diarrhoea.

Subgroups	Adjusted OR (95%CI)	P-value	P for interaction
Age (year)			0.242
<60 years	1.50 (1.04–2. 15)	0.030	
≥60 years	1.27 (0.84–1.93)	0.251	
Sex			0.009
Male	2.76 (1.70–4.47)	<0.001	
Female	0.92 (0.63–1.35)	0.668	
Race			0.855
Mexican	1.50 (0.79–2.85)	0.206	
American			
Non-Hispanic White	1.26 (0.89–1.80)	0.186	
Non-Hispanic Black	1.53 (0.79–2.98)	0.197	
Others	1.76 (0.84–3.67)	0.126	
CVD			0.867
Yes	1.31 (0.39–4.38)	0.644	
No	1.35 (1.01–1.79)	0.041	
Diabetes			0.430
Yes	2.08 (1.00–4.35)	0.051	
No	1.30 (0.96–1.76)	0.090	
Hypertension			0.623
Yes	1.65 (1.10–2.46)	0.017	
No	1. 18 (0.82–1.69)	0.361	

BRI, body roundness index; OR, odds ratio; CI, confidence interval; CVD, cardiovascular disease.

### 3.4 BRI is stronger than ABSI, VAI, BMI and WC in predicting chronic diarrhoea

**Figure 3:**
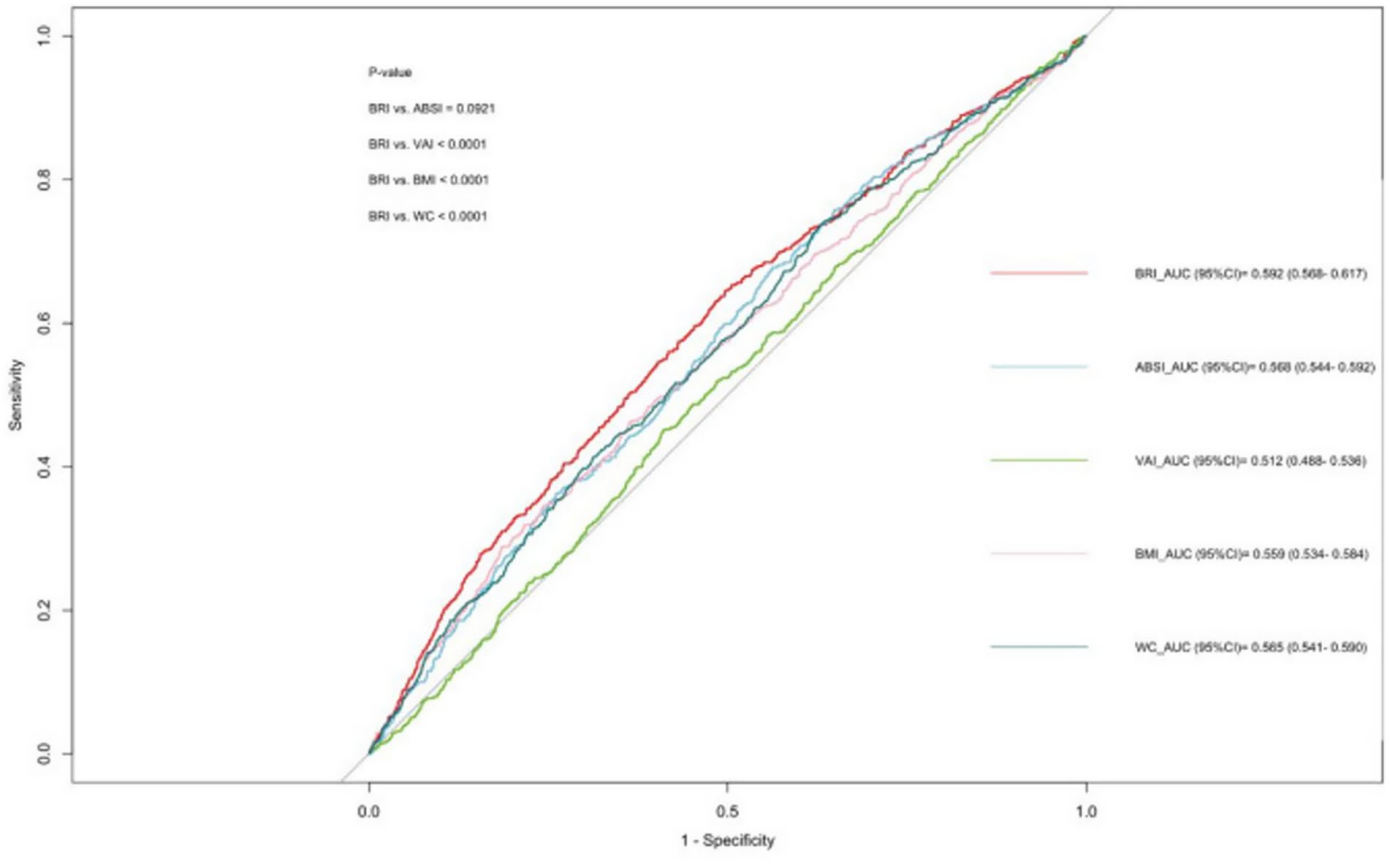
Anthropometric indicators for predicting chronic diarrhoea.

The ROC curve analysis showed that BRI and ABSI outperformed VAI, BMI, and WC in predicting chronic diarrhoea (Figure 3). The area under the curve (AUC) for BRI was 0.592, significantly higher than ABSI (0.568), BMI (0.559), WC (0.565), and VAI (0.512), indicating BRI’s enhanced classification accuracy in distinguishing chronic diarrhoea cases from non-cases. Although ABSI exhibited a lower AUC than BRI, it demonstrated higher sensitivity (0.678 vs. 0.646 for BRI), suggesting greater capability in identifying true positive cases. Conversely, BRI showed better specificity (0.503 vs. 0.437 for ABSI), supporting its utility in ruling out non-cases. Collectively, both BRI and ABSI outperformed traditional anthropometric indices (VAI/BMI/WC) in diagnostic efficacy, with BRI achieving marginally superior overall performance (AUC/specificity) and ABSI excelling in sensitivity (Table 5).

**Table 5:** Anthropometric indicators for predicting chronic diarrhoea.

Test	AUC	95%CI low	95%CI upp	Cutoff value	Specificity	Sensitivity
BRI	0.592	0.568	0.617	4.825	0.503	0.646
ABSI	0.568	0.544	0.592	0.080	0.437	0.678
VAI	0.512	0.488	0.536	2.515	0.589	0.452
BMI	0.559	0.534	0.584	33.335	0.816	0.287
WC	0.565	0.541	0.590	91.950	0.370	0.737

BRI, body roundness index; ABSI, a body shape index; VAI, visceral adiposity index; BMI, body mass index; WC, waist circumference; AUC, area under the curve; CI, confidence interval.

The area under the curve (AUC) values demonstrate the diagnostic accuracy of each indicator: BRI (Body Roundness Index, dark blue line, AUC = 0.592, 95% CI: 0.568–0.617), ABSI (A Body Shape Index, red line, AUC = 0.568, 95% CI: 0.544–0.592), VAI (Visceral Adiposity Index, orange line, AUC = 0.512, 95% CI: 0.488–0.536), BMI (Body Mass Index, green line, AUC = 0.559, 95% CI: 0.534–0.584), and WC (Waist Circumference, purple line, AUC = 0.565, 95% CI: 0.541–0.590). Higher AUC values indicate better discriminatory ability. The diagonal reference line represents random chance (AUC = 0.5). Statistical significance between curves was assessed using the DeLong test (*p* < 0.010).

## 4 Discussion

This study elucidated the association between visceral adiposity index (BRI/ABSI) and chronic diarrhoea disease by population level analysis method. The results showed that both indices were significantly and positively associated with chronic diarrhoea. After stratified multivariate logistic regression analysis and adjustment for all relevant covariates, a 1-unit increase in BRI was associated with a 13% increase in the risk of chronic diarrhoea (OR = 1.13, 95% CI: 1.08–1. 19), whereas a 0.01-unit increase in ABSI corresponded to a 35% increase in the risk of chronic diarrhoea (OR = 1.35, 95% CI: 1.01–1.80). These findings suggest that BRI and ABSI are significant independent predictors of chronic diarrhoea. The most striking finding in our study was that there was a significant gender difference in the association of ABSI with chronic diarrhoea (male OR = 2.76 vs. female OR = 0.92, *p* interaction = 0.009), whereas the association of BRI with chronic diarrhoea did not differ significantly between genders (*p* interaction = 0.737). This gender-specific difference reflects the originality of this study. From the perspective of physiological mechanisms, males secrete 5–10% more bile acids than females, and androgens promote CYP27A1 gene expression through the JNK signaling pathway (32, 33), significantly increases bile acid synthesis (34–36). Bile acids activate TGR5 receptors on intestinal smooth muscle (37, 38), enhance peristalsis strength and frequency, promote peristalsis acceleration and thus diarrhoea (39–43). Men tend to accumulate visceral adiposity (VAT), which is associated with increased metabolic syndrome and cardiovascular risk (44–47), expressed as elevated serum lipopolysaccharide (LPS) levels and decreased expression of tight junction proteins (ZO-1, Occludin and Claudin-3) (48, 49), and widening of cell gaps. These changes lead to increased permeability of the intestinal barrier, which in turn triggers diarrhoea (50–54). In contrast, estrogen in women can increase Nrf2-associated signaling pathways by inhibiting pathways such as NF-κB inflammation (55), increases beneficial bacteria and thus provides a protective effect on the intestinal tract (56–58). Consistent with previous studies we found a strong association of ABSI with chronic diarrhoea in men (OR = 2.76) reflecting the tendency for men to have a higher proportion of visceral fat distribution.

Another noteworthy result was the complementary value of BRI and ABSI in the diagnosis of chronic diarrhoea. BRI outperformed ABSI in overall predictive accuracy (AUC: 0.592 vs. 0.568), but ABSI performed better in sensitivity (0.678 vs. 0.646), while BRI was stronger in specificity (0.503 vs. 0.437). This finding suggests that the ABSI may be more valuable and the BRI may be more useful in clinical screening of high-risk populations when excluding non-cases. This complementarity further confirms that the two indices measure different aspects of abdominal fat distribution: the BRI focuses more on overall body fat distribution, whereas the ABSI emphasizes the geometric properties of body shape.

In the present study, it was found that high BRI and ABSI values represent accumulation of abdominal fat, and excessive abdominal fat secretes inflammatory factors such as alpha (TNF-α), interleukin 6 (IL-6), leptin, and others (59). TNF-α, IL-6 and leptin through common signaling pathways such as JAK–STAT (60–62), NF-κB and MAPK pathways (63–67), all three signaling pathways work together to trigger immune responses and inflammatory cascades, and this “leaky gut” phenomenon may be directly involved in the development of functional diarrhoea (68–70). Secondly, pro-inflammatory factors such as TNF-α, IL-6 and IL-1β stimulate degranulation of intestinal mast cells, releasing biologically active substances such as histamine and 5-hydroxytryptophan, which are potent stimulants of intestinal motility and promote peristalsis (71, 72). Finally, at the base of the brain-gut axis, excess fatty acids in obese individuals cause chronic inflammation of the hypothalamus (73–78), impairment of its ability to regulate the fine balance of intestinal motility, along with an abnormal increase in neurotransmitters such as intestinal 5-hydroxytryptamine and the formation of abnormal signaling loops through the vagus nerve, leading to over-excitement and disordered high-frequency contraction of the intestinal nervous system, which is ultimately manifested as recurrent diarrhoea (79, 80).

Our findings suggest that high ABSI and BRI values are mainly associated with several specific types of chronic diarrhoea. First, functional diarrhoea (FDr) and diarrhoeal irritable bowel syndrome (IBS-D) may be closely associated with visceral fat accumulation, which is mainly mediated through visceral fat-triggered low-grade inflammation and gut microbiome dysbiosis (15, 16). Secondly, high ABSI and BRI values were significantly associated with bile acid diarrhoea, which may be due to increased bile acid synthesis and abnormal bile acid metabolism in obese individuals. Third, diarrhoea due to small intestinal bacterial overgrowth syndrome (SIBO) is more common in people with a high visceral adiposity index, which is associated with altered obesity-associated bacterial communities (81–83). Notably, the association of diarrhoea with visceral fat found in the present study was predominantly in the form of altered fecal character (Bristol scale type 6–7) rather than inflammatory bowel disease (IBD)-associated diarrhoea, which was particularly evident in males (OR = 2.76), suggesting that the distribution of abdominal fat has an important influence on functional bowel disease.

The present study has several methodological strengths: the use of the NHANES population-level dataset ensured the robustness of the analysis; nationally representative sampling enhanced the validity of epidemiologic inferences; and a rigorous multivariate confounding control scheme enhanced the validity of causal inferences. However, limitations remain (8): self-reported bowel habits may be subject to recall bias (3); categorization based on the “usual or most common” stool type in the Bowel Health Questionnaire may not be fully consistent with the Rome IV criteria for functional diarrhoea (84); the cross-sectional design did not allow for the establishment of a causal relationship between bowel patterns and ABSI/BRI; and (27) the 2005–2010 data collection period may have been time-limited. May have been time-limited.

In conclusion, our investigation pioneers the use of nationally representative NHANES data to establish BRI and ABSI as crucial predictors of chronic diarrhoea. Our findings revealed that every one-unit elevation in BRI corresponded to a 13% increased likelihood of developing chronic diarrhoea, whereas each 0.01-unit rise in ABSI was linked to a 35% greater risk. Notably, BRI outperformed conventional anthropometric indicators in predictive accuracy, while ABSI exhibited pronounced sex-specific effects, particularly among male participants. These results underscore the pivotal influence of visceral adiposity on gastrointestinal dysfunction and chronic diarrhoeal disorders.

## 5 Conclusion

The objective of this study was to investigate the association between two novel abdominal fat indicators (ABSI and BRI) and chronic diarrhoea using nationally representative NHANES data (2005–2010). For the first time, we have identified ABSI and BRI as potentially useful clinical predictors of chronic diarrhoea.

## Data Availability

Publicly available datasets were analyzed in this study. This data can be found here: the data sets used in this study were all publicly available from NHANES (https://www.cdc.gov/nchs/nhanes/index.htm).

## Footnotes

^1^ https://www.cdc.gov/nchs/nhanes/

^2^ https://www.r-project.org/

## Glossary

ABSI: A body shape index
AUC: Area under the curve
BHQ: Bowel health questionnaire
BMI: Body mass index
BRI: Body roundness index
BSFS: Bristol stool form scale
CI: Confidence interval
CVD: Cardiovascular disease
DBP: Diastolic blood pressure
FDr: Functional diarrhoea
FPG: Fasting plasma glucose
Hb: Hemoglobin
HbA1c: Hemoglobin A1c
IBS-D: diarrhoeal irritable bowel syndrome
IBD: Inflammatory bowel disease
IL-6: Interleukin-6
LPS: Lipopolysaccharide
MAPK: Mitogen-activated protein kinase
NCHS: National Center for Health Statistics
NF-κB: Nuclear Factor kappa B
NHANES: National Health and Nutrition Examination Survey
OGTT: Oral glucose tolerance test
OR: Odds ratio
PIR: Poverty income ratio
Q1: First quartile
Q2: Second Quartile
Q3: Third Quartile
Q4: Fourth Quartile
RCS: Restricted cubic spline
ROC: Receiver operating characteristic
SBP: Systolic blood pressure
SIBO: Small intestinal bacterial overgrowth
TC: Total cholesterol
TG: Triglycerides
TNF-α: Tumor necrosis factor-alpha
VAI: Visceral Adiposity Index
VAT: Visceral Adipose Tissue
WC: Waist Circumference
